# Current Status of Cardiovascular Disease According to the Duration of Hypertension in Korean Adults

**DOI:** 10.5334/gh.1201

**Published:** 2023-05-10

**Authors:** Byoung Geol Choi, Jung Boone Kim, Seung-Woon Rha, Min Woo Lee, Michael S. Lee, Suhng Wook Kim, Sunghoi Hong

**Affiliations:** 1 Cardiovascular Research Institute, Korea University, Seoul, Korea; 2 Department of Integrated Biomedical and Life Sciences, Korea University Graduate School, Seoul, Korea; 3 Cardiovascular Center, Korea University Guro Hospital, Seoul, Korea; 4 Research Institute of Health Science, Korea University, Seoul, Korea; 5 UCLA Medical Center, Los Angeles, California, USA; 6 School of Health and Environmental Science, College of Health Sciences, Korea University, Seoul, Korea; 7 School of Biosystems and Biomedical Sciences, Korea University Graduate School, Seoul, Korea

**Keywords:** hypertension, blood pressure, prevalence, target blood pressure

## Abstract

**Background:**

Today, medical technology and healthcare advances have led to an increased life expectancy; however, the prevalence of chronic diseases such as hypertension, diabetes mellitus, stroke, and cardiovascular events is continuously rising. In particular, hypertension is a crucial factor in cardiovascular and cerebrovascular diseases, and it is known that prevention and management are essential.

**Objectives:**

This study investigates the prevalence and management of hypertension in Korean adults and evaluates its correlation with the risk of cardiovascular disease (CVD) and stroke.

**Method:**

The Korean National Health and Nutritional Examination Survey (KNHANES) database was utilized for this study (https://knhanes.cdc.go.kr). The subjects of this survey were sampled to represent the entire population of Korea. The study aims to assess the risk of CVD and stroke according to the duration of hypertension. We also examined the impact of hypertension control on the risk of CVD and stroke. This study is a retrospective cross-sectional study, so future risks cannot be assessed, but only the disease status at the same time point.

**Results:**

A total of 61,379 subjects were enrolled in the KNHANES database, representing Korea’s population of 49,068,178 subjects. The prevalence of hypertension was 25.7% (9,965,618 subjects) of the total population. The prevalence of hypertension increased rapidly with the age of the population. As the duration of hypertension increased, the risks of CVD and stroke also increased. When hypertension lasts longer than 20 years, ischemic heart disease, myocardial infarction, and stroke prevalence were 14.6%, 5.0%, and 12.2%, respectively. However, achieving a target blood pressure (BP) goal below 140/90 mmHg reduced the risk of all CVD and stroke by nearly half. Nevertheless, fewer than two-thirds of patients in Korea with hypertension achieved this targeted blood pressure goal.

**Conclusions:**

Our study confirmed that the prevalence of hypertension in Korean adults was higher than a quarter but also showed that the risk of CVD and stroke was significantly reduced by achieving optimal blood pressure control. Based on these results, policy efforts are needed to reach the target BP and improve the treatment rates for hypertension in Korea.

## Introduction

Life expectancy worldwide has increased steadily for nearly 200 years, along with advances in medicine and health systems [1,2]. However, chronic diseases increase rapidly as human life expectancy improves. Chronic diseases are generally progressive, long-term medical conditions, for example, hypertension, diabetes, heart disease, and stroke [2].

Especially hypertension is the most common condition seen in primary care, and its progress often asymptomatically (the so-called silent killer) [3]. It is known to be closely related to a higher risk of cardiovascular events, renal failure, and death [1,2,3]. For example, cardiovascular disease (CVD) is known to be the leading cause of death in the America and raised blood pressure accounts for over 50% of CVD [4]. Therefore, the World Health Organization (WHO) highlights the prevention and control of hypertension, the most common and central point of entry for the primary care management of CVD [3,5]. For the diagnosis and management of hypertension, it is essential to track and monitor the prevalence and management status of the population.

This study evaluated the prevalence of hypertension, its treatment, and the effects of hypertension on cardiovascular disease and stroke risk in Koreans. The results of this study expected that it would support a comprehensive strategic approach to hypertension prevention and blood pressure management.

## Method

The design of the Korean National Health and Nutritional Examination Survey (KNHANES) has been previously described [6,7]. This survey includes a health interview, behavioral health interview, health examination, and nutrition assessment. The KNHANES is a publicly available database (https://knhanes.cdc.go.kr) that includes detailed information on socioeconomic status, health and dietary behavior, quality of life, medication use, anthropometry, and biochemical profiles. It is used for random sampling of the health and nutritional status of Korean adults. Training healthcare professionals (nurses and technicians) collected data using standardized case report forms and protocols. The KNHANES uses a complex, multi-stage probability sample design. The sample represents the total non-institutionalized civilian population of Korea. Therefore, a three-stage sample design is used for the KNHANES.

The primary sample units (PSUs) are selected from a sampling frame of all census blocks or resident registration addresses. Each PSU consists of approximately 50–60 households. Following the PSU selection, all dwelling units in the PSU are listed, and 20 households are selected through the field survey for household screening. The final selection stage occurs in the household, where all members aged one year and over are selected to participate. Approximately 10,000 persons are sampled in total in all 192 PSUs per year. To produce unbiased cross-sectional estimates for the entire Korean population, the sample data can be inflated to the level of the population from which the sample is drawn. The most crucial consideration in analyzing KNHANES data involves considering the survey design. Survey sample weights should be used, and the complex survey design must be accounted for in estimating variance [3]. The proper usage of sampling weights ensures that calculated estimates are truly representative of the Korean population. The sampling weights for each sample person are the product of three factors: the reciprocal of the probabilities of selection (PSU, household); an adjustment for non-response (household, person); and a post-stratification factor to make the resulting survey estimates for age, sex, metropolitan area, or province category approximately equal to the total population of Korea. Sampling weights can be averaged over the sampled years to analyze samples over multiple years [8].

**Data source and population:** A total of 61,379 subjects were enrolled in the KNHANES database from 2007 to 2014, representing 49,068,178 subjects. For the analysis of this study, we excluded adolescents under the age of 18 or elderly subjects over 80 years of age. The final analysis included 45,811 subjects representing 38,755,871 people. Patients with hypertension were stratified according to the duration of hypertension into groups of less than one year, one to five years, six to 10 years, 11 to 15 years, 16 to 20 years, and more than 20 years.

**Blood pressure measurement (https://knhanes.cdc.go.kr):** Blood pressure (BP) was measured with a mercury sphygmomanometer with an appropriately sized cuff after the subject sat quietly for five minutes with the right arm supported at the level of the heart. In determining the mean BP for individuals, the first BP was used if only one measurement had been taken. The second BP was used if two readings had been taken. The second and third values were averaged when available. The same measurement instruments (Baumanometer; WA Baum Co., New York, NY, USA) were used. Quality control activities for these BP measurement methods were observed during the survey.

**Study definition:** We defined hypertension as follows: 1) a BP 140/90 mmHg or higher, 2) a patient diagnosed with hypertension by the clinician, or 3) a clinician-prescribed antihypertensive medication. The target BP (TBP) goal was less than 140/90 mmHg or less than 130/80 mmHg in those patients with diabetes or chronic kidney disease [3,9,10,11].The achievement of target BP (a TBP) is defined as a control under the TBP after the awareness of hypertension. The duration of hypertension is defined as the period from the time the patient was diagnosed or recognized with hypertension to the time of study enrollment.

**Statistical analysis:** For continuous variables, differences between the two groups were evaluated by an unpaired t-test or a Mann-Whitney rank test. Data were expressed as a mean ± standard error. For discrete variables, differences were expressed as counts and percentages and analyzed with χ2 or Fisher’s exact test between the groups as appropriate. Weights based on the complex KNHANES sampling design were used in all statistical analyses to avoid biased estimates. A crude and multivariable logistic regression model was performed to adjust for potential confounders. A two-tailed p-value less than 0.05 was considered to be statistically significant. All data analyses were performed with SPSS (version 20.0, SPSS-PC, Inc., Chicago, Illinois).

## Result

**Figure 1:**
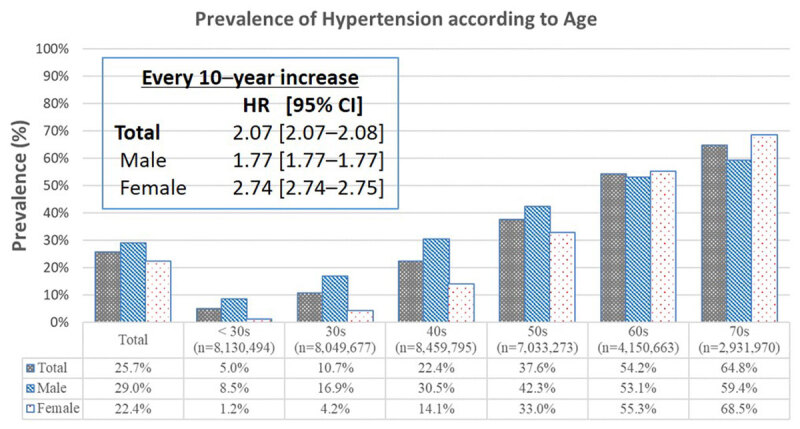
Trends in hypertension according to age in the Korean population by a crude logistic regression modelanalysis.

Of the 38,755,871 (n = 45,811) subjects, 19,363,419 (n = 19,976) were male, and 19,392,453 (n = 25,835) were female. The prevalence of hypertension was 25.7% (9,965,618 subjects) of the total population, with 29.0% (5,616,349 subjects) being male and 22.4% (4,349,270 subjects) being female. The hypertension sex ratio was 1.3:1 (Figure 1). The prevalence of hypertension increased from 5.0% to 64.8% as patient age increased from the 20s to the 70s. The mean age of the hypertensive population was 55.8 ± 13.7 years, and the male patients were younger than the female patients (52.1 ± 13.9 years vs. 60.5 ± 11.8 years, *p* < 0.01). The hypertension group has significantly poorer baseline characteristics than the non-hypertension group (Table 1). Whenever, in the logistic regression analysis, the ages increase by 10 years as compared to the 20s group, the risk of hypertension increases 2.07 (95% CI: 2.07–2.08, *p* < 0.01) times of the whole population, 1.77 (95% CI: 1.77–1.77, *p* < 0.01) times for males, and 2.74 (95% CI: 2.74–2.75, *p* < 0.01) times for females respectively (Figure 1).

**Table 1:** Baseline characteristics according to the presence of the hypertension.


VARIABLES, %	TOTAL		MALE		FEMALE	
		
	HTN (N = 13,386) (9,965,619)	NON-HTN (N = 32,425) (28,790,253)	HTN (N = 6,509) (5,616,349)	NON-HTN (N = 13,467) (13,747,070)	HTN (N = 6,877) (4,349,270)	NON-HTN (N = 18,958) (15,043,183)

**Age, years**	55.8 ± 13.7	40.1 ± 14.1	52.1 ± 13.9	39.8 ± 14.2	60.5 ± 11.8	40.4 ± 14

**Sex (male/female)**	1.3	0.9	–	–	–	–

Male	43.6 %	52.3 %	–	–	–	–

Female	56.4 %	47.7 %	–	–	–	–

**Blood pressure, mmHg**			–	–	–	–

Systolic	134 ± 16	110 ± 11	133 ± 15	113 ± 10	134 ± 17	108 ± 11

Diastolic	84 ± 12	73 ± 8	87 ± 11	75 ± 8	81 ± 11	70 ± 8

**Heart rate, bpm**	69 ± 10	70 ± 9	69 ± 11	68 ± 9	70 ± 9	71 ± 9

**BMI, Kg/m** ^2^	25.1 ± 3.4	23.1 ± 3.3	25.1 ± 3.3	23.6 ± 3.1	25.1 ± 3.6	22.6 ± 3.3

**Diabetes**	19.3 %	4.7 %	18.4 %	5.8 %	20.5 %	3.7 %

**Impaired fasting glucose**	26.0 %	12.8 %	28.8 %	15.5 %	22.5 %	10.4 %

**Arrhythmia**	2.1 %	1.1 %	2.2 %	1.3 %	2.0 %	0.8 %

**Dyslipidemia**	50.8 %	26.8 %	51.9 %	34 %	49.3 %	20.2 %

**Ischemic heart disease**	3.9 %	0.8 %	3.4 %	1.0 %	4.5 %	0.7 %

Myocardial infarction	1.3 %	0.3 %	1.5 %	0.5 %	1.1 %	0.2 %

**Angina pectoris**	2.7 %	0.6 %	2.1 %	0.6 %	3.6 %	0.5 %

**Stroke**	3.6 %	0.5 %	3.5 %	0.6 %	3.7 %	0.4 %

Disability	2.3 %	0.3 %	2.2 %	0.4 %	2.4 %	0.2 %

**Chronic renal failure**	0.9 %	0.1 %	0.6 %	0.1 %	1.1 %	0.2 %

**Thyroid disease**	3.1 %	2.7 %	0.9 %	0.7 %	5.8 %	4.5 %

**Pulmonary disease**	17.7 %	6.7 %	20.8 %	8.9 %	13.6 %	4.7 %

Obstructive	9.3 %	3.6 %	12.3 %	5.6 %	5.4 %	1.8 %

Restrictive	8.3 %	3.1 %	8.4 %	3.3 %	8.2 %	3.0 %

**Anemia**	6.6 %	7.2 %	3.8 %	1.7 %	10.3 %	12.2 %

Cancer	3.3 %	1.8 %	2.6 %	1.4 %	4.2 %	2.1 %

Depression disorder	5.1 %	4.0 %	2.4 %	2.0 %	8.5 %	5.9 %

Current alcohol	67.9 %	75.7 %	82.3 %	82.4 %	49.3 %	69.5 %

Current smokers	31.6 %	29.6 %	51.3 %	52.1 %	6.0 %	9.0 %


Data are presented as percentage, % or mean ± standard deviation. HTN: hypertension, BMI: body mass index.

The prevalence of ischemic heart disease (IHD) and stroke in the whole population is 1.6% (627,213 subjects) and 1.3% (503,847 subjects), respectively. As compared to the non-hypertension group, the hazard ratio of IHD and stroke was 4.70 (95% CI: 4.68–4.73, *p* < 0.01) and 7.42 (95% CI: 7.37–7.46, *p* < 0.01) times higher in the hypertensive group, respectively, by simple logistic regression analysis. Furthermore, the hazard ratio of IHD and stroke are 1.72 (95% CI: 1.71–1.73, *p* < 0.01) times higher and 2.78 (95% CI: 2.77–2.80, *p* < 0.01) times higher, respectively, after adjustment by multiple logistic regression analysis for sex and age.

**Figure 2:**
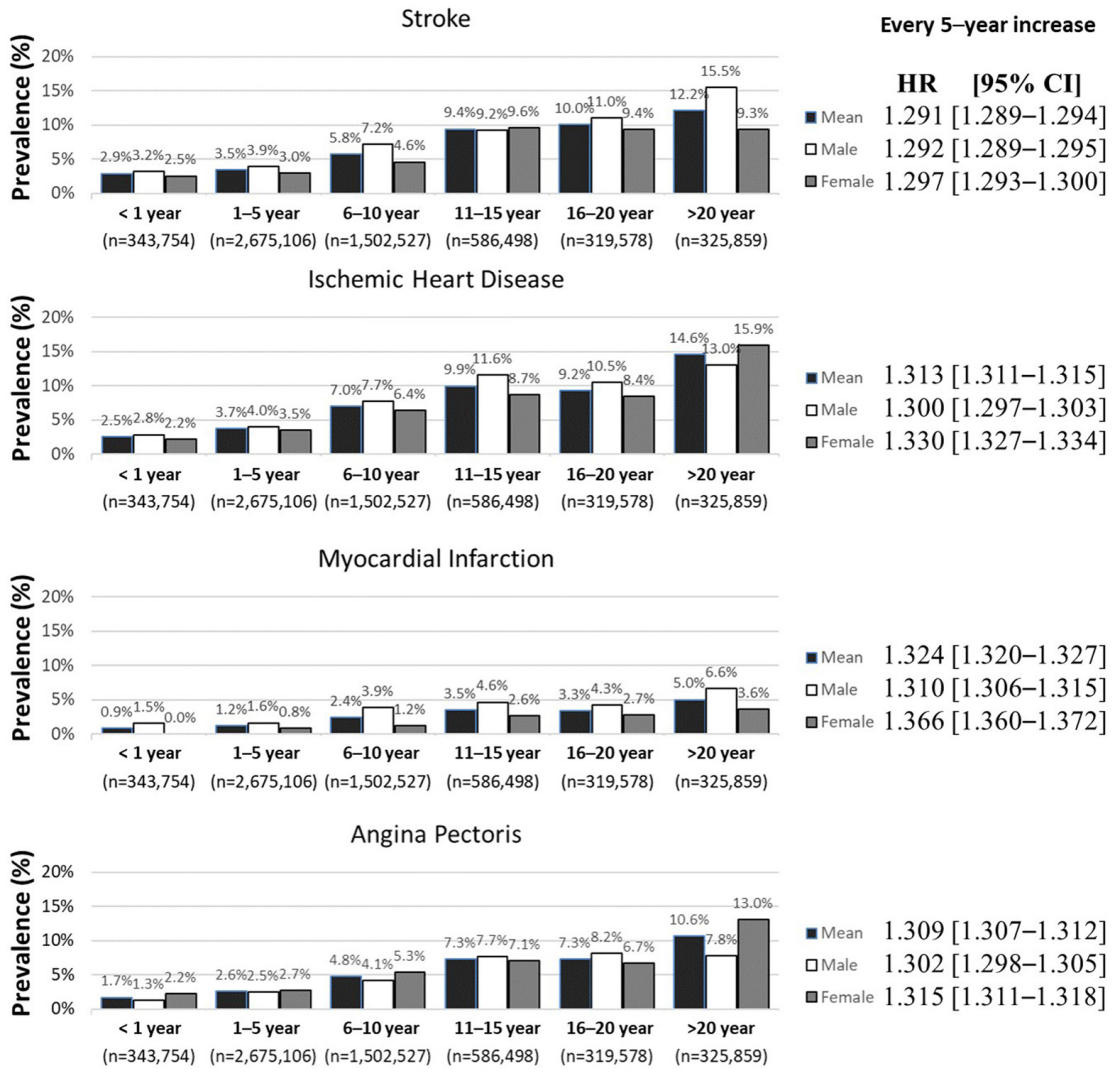
Trends in cardiovascular disease according to the duration of hypertension in the Korean population by a crude logistic regression model analysis. HR: hazard ratio, CI: confidence interval.

Compared to the hypertensive group diagnosed within a year, if hypertension increases to five years, the risk for all CVDs, such as IHD, myocardial infarction (MI), angina pectoris (AP), and stroke increases to nearly 30%, regardless of sex. When the duration of hypertension was greater than 20 years, the prevalence of IHD, MI, AP, and stroke was 14.6%, 5.0%, 10.6%, and 12.2%, respectively (Figure 2).

**Figure 3:**
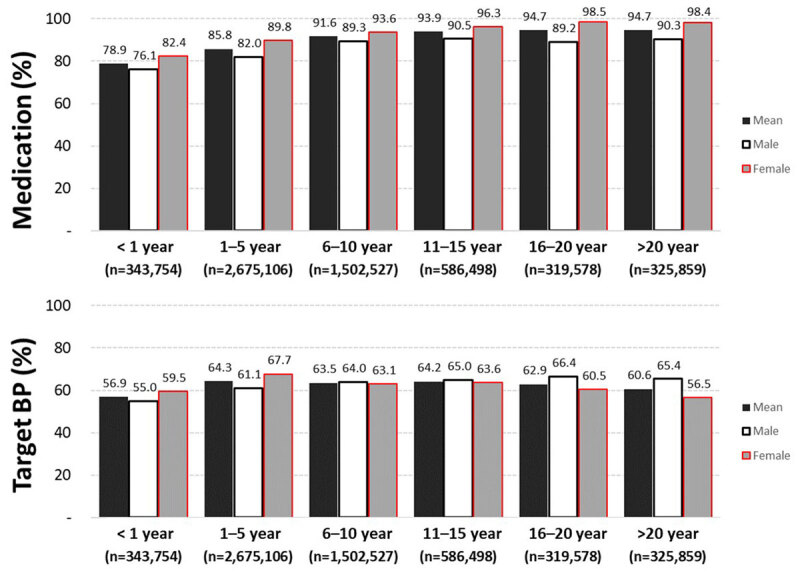
Treatment of hypertension and achievement of targeted blood pressure (BP) according to the duration of hypertension in the Korean population.

Figure 3 shows the treatments for hypertension and the TBP according to the duration of hypertension. Treatment for hypertension, such as medication and improvement of diet and lifestyle, was 78.9% within one year of diagnosis, but it was 94.7% in hypertension survivors after 20 years. An achievement rate of a TBP was 56.9% within the first year of diagnosis and 60.6% in hypertension survivors after 20 years.

Table 2 shows the impact of achieving a targeted BP on IHD or stroke in the hypertensive population. In multiple logistic regression, compared to those who did not achieve the BP target, the targeted BP achievement group significantly reduced the risk of IHD, MI, AP, and stroke by more than half, and this risk was reduced by nearly 30% even after adjusting for sex, age, and glucose control via hemoglobin A1c.

**Table 2:** Impact of achieving a target blood pressure on IHD or stroke in the hypertensive population by unadjusted/adjusted logistic regression analysis for sex, age, and glucose control via hemoglobin A1c level.


PREVALENCE, %	TOTAL (N = 13,386) (9,965,619)	ATBP (N = 7,444) (5,967,772)	NON-ATBP (N = 5,942) (3,997,847)	P VALUE	UNADJUSTED HR [95% CI]	ADJUSTED HR [95% CI]

**Stroke**	3.6 %	2.3 %	5.5 %	<0.001	0.408 [0.405–0.411]	0.628 [0.622–0.634]

**Ischemic heart disease**	3.9 %	2.5 %	5.9 %	<0.001	0.414 [0.411–0.417]	0.723 [0.717–0.729]

**Myocardial infarction**	1.3 %	0.9 %	2.0 %	<0.001	0.448 [0.443–0.453]	0.693 [0.683–0.703]

**Angina pectoris**	2.7 %	1.7 %	4.2 %	<0.001	0.399 [0.395–0.402]	0.706 [0.699–0.713]


aTBP: achieve target blood pressure (< 90/140 mmHg), HR: hazard ratio, CI: confidence interval.

Table 3 shows a predictor for the failure of achieving TBP in the hypertensive population. In multiple logistic regression analysis, anemia, prior stroke, aging, men, diabetes mellitus, and standard range of body mass index (18 to 24) are significant predictors for the failure of achieving TBP. In contrast, the patients with pulmonary disease, long-standing hypertension, prior IHD including MI and angina, and dyslipidemia are well-achieving TBP.

**Table 3:** Predictors for the failure of achieving target blood pressure in the hypertensive population by multiple logistic regression analysis.


PREDICTORS	HR (95% CI)	P VALUE

**Anemia**	1.179 (1.177–1.182)	<0.001

**Prior stroke**	1.109 (1.106–1.112)	<0.001

**Age (per 5-year increment)**	1.085 (1.084–1.085)	<0.001

**Sex (male vs. female)**	1.051 (1.05–1.0530)	<0.001

**Diabetes mellitus**	1.036 (1.035–1.038)	<0.001

**Body mass index: BMI, kg/m2**		<0.001

18 ≤ BMI < 24	Control	

BMI < 18	0.918 (0.911–0.925)	<0.001

BMI ≥ 24	0.943 (0.942–0.944)	<0.001

**Pulmonary disease**	0.954 (0.952–0.955)	<0.001

**Duration of hypertension (per 5-year increment)**	0.946 (0.946–0.947)	<0.001

**Ischemic heart disease**	0.945 (0.943–0.948)	<0.001

**Dyslipidemia**	0.928 (0.927–0.929)	<0.001


HR: hazard ratio, CI: confidence interval. Variables in the table were selected as well-known variables correlated with hypertension and presented in descending order of hazard ratio.

## Discussion

The main findings of our study are as follows:

1. The prevalence of hypertension increases rapidly with the increased age of the population. The prevalence of hypertension is incredibly high in men, but the rates are increasing more rapidly in women.
2. Our research shows that CVD and stroke risks increase epidemiologically when the duration of hypertension increases. For example, when the duration of hypertension in Koreans is increased by five years, the risk of CVD and stroke increases by nearly 30%.
3. However, we confirmed that achieving a targeted BP below 140/90 mmHg can prevent nearly half of all CVD and stroke risks in hypertensive patients.
4. Nevertheless, fewer than two-thirds of those hypertensive patients in Korea achieve this targeted blood pressure.
5. Anemia, prior stroke, aging, men, diabetes mellitus, and standard range of body mass index (18 to 24) are significant predictors for the failure of achieving TBP.

According to OECD resources (www.oecd.org), South Korea is one of the countries that lead in having a rapidly aging segment of society, and the prevalence of chronic diseases is also increasing. The present study delineates the prevalence of Korea’s hypertension and its management status. In addition, this study evaluates the effect of the duration of hypertension on the occurrence of stroke and cardiovascular disease. In previous studies, there have been many reports on long-term prognosis analysis of the correlation between hypertension and CV risk. However, as in our study, studies that quantitatively analyzed CV risk during the prevalence of hypertension over 20 years were limited.

In our study, Korean adults in their 50s diagnosed with hypertension are one-third of the population at 37.6%. If they live out their expected Korean lifespan of 83.5 years (www.oecd.org, 2020), they will have to deal with hypertension for more than 20 years. Therefore, patients with hypertension may be exposed to high-incidence risks of 14.6% for CVD and 12.2% for stroke over 20 years of the hypertension period. If hypertension in Koreans persists for five years, CVD and stroke risk increase by nearly by 30%. As shown in Figure 1, throughout the entire lifespan, the prevalence of hypertension is notably higher in men (29.0 % vs. 25.7%), but rates of hypertension increase more rapidly in women (x1.77 vs. x2.74). Based on these findings, a policy of hypertension management for Koreans that reflects life-cycle characteristics, such as sex and age, may be required.

Our study shows that 78.9% of patients diagnosed with hypertension within a year and 94.7% of patients who had survived over 20 years of hypertension received antihypertensive therapy. However, the achievement of a targeted BP below 140/90 mmHg was less than two-thirds over the whole hypertensive period. Surprisingly, our results show that achieving a TBP can reduce the risk of future CVD and stroke by nearly one-half. So, strictly achieving a targeted BP requires a continuous and meticulous BP monitoring system. Many randomized controlled trials have proven the benefits of antihypertensive therapy to improve clinical outcomes [9,12,13]. The Joint National Committee (JNC)-8 and the Korean Society of Hypertension defined hypertension as a BP of 140/90 mmHg or higher, and they recommend a TBP less than that except in the elderly (systolic BP of less than 150 mmHg) [9,14,15,16]. Furthermore, the 2017 American College of Cardiology/American Heart Association (ACC/AHA) hypertension guidelines have recently recommended that the TBP goal be less than 130/80 mmHg [10]. Guidelines suggest that high BPs should be treated earlier with lifestyle changes and in some patients with medication with a goal of 130/80 mmHg instead of 140/90 mmHg. Lee et al., using the data from the National Health Insurance System and National Health Screening Cohort, evaluated the association between BP control and long-term cardiovascular outcomes in Koreans, including the composite of cardiovascular death, MI, heart failure, and stroke (median follow-up of 11.0 years) [6]. They reported that the group following the 2017 ACC/AHA hypertension guidelines had a risk reduction of 23% in long-term significant cardiovascular outcomes compared to the JNC-8 group. The definition of an optimal BP target can be controversial. There is already abundant evidence demonstrating the benefits of reducing blood pressure and maintaining it at a targeted level below 140/90 mmHg for improving important health outcomes in hypertensive patients [9,12,13].

The results of our study demonstrate a high prevalence of hypertension in Korean adults and its risks of complications. In addition, it is noted that when the treatment target for BP is reached, the risk of complications is reduced. Nevertheless, the low rates and achievements of target BPs have limits. Academia and the government’s consistent policy efforts could improve this. Hypertension and its complications, such as CVD and stroke risk, contribute to premature death and poor quality of life.

Furthermore, hypertension creates cost problems in management because it is the single most significant contributor to the socioeconomic burden of disease worldwide [3,5,17,18]. Therefore, hypertension prevention and early treatment are essential for managing CVD and stroke risk patients. Such approaches can reduce the socioeconomic burden caused by the disease. For example, the Korean government is trying to manage chronic diseases via a policy called the ‘National Chronic Disease Management in Primary Care-Hypertension and Diabetes Care,’ according to a report by the Korea Centers for Disease Control and Prevention (KCDC). In 2019, they set goals such as ‘improvement and maintenance of treatment sustainability’ for hypertensive patients. They are expected to improve the national health insurance budget, which is $2.1 billion annually.

This study had some limitations. Our research was conducted with representative samples and surveys of the entire population. Also, only survivors were included at the time of the investigation. The risk of death from disease was not assessed. This study is a cross-sectional study. Thus, we do not simply conclude the causality of the findings, especially regarding TBP and CVD risk. This study is representative of the population, and there may be uncertainty in quantifying future risks. Lifestyle modification was not assessed in this study. Lifestyle modification is effective in lowing BP. This study may have other unsettled confounders, such as other pharmacotherapy, including statin and antiplatelet therapy. TBP was defined as being less than 140/90 mmHg. This optimal TBP is controversial, but it was the definition used at the time of enrollment of the study subjects.

In conclusion, based on our present study, we can predict that the duration of hypertension will increase as human life expectancy improves. Furthermore, the longer the duration of hypertension, the greater the risk of CVD and stroke. Therefore, policy efforts are required to reduce the duration of hypertension by delaying its development, achieving a TBP, and improving the treatment of hypertensive patients. Especially hypertension patients with anemia, prior stroke, elderly, men, diabetes mellitus, and the normal range of body mass index group requires active treatment and closed follow-up to achieve TBP. Therefore, our study results urge us to prioritize hypertension prevention and control to improve their populations’ health and well-being and reduce CVD.

## Data accessibility Statement

All data can be checked by sending an email to correspondence.
